# Controlled Drug Release and Cytotoxicity Studies of Beta-Lapachone and Doxorubicin Loaded into Cyclodextrins Attached to a Polyethyleneimine Matrix

**DOI:** 10.3390/ijms21165832

**Published:** 2020-08-14

**Authors:** Agata Kowalczyk, Artur Kasprzak, Magdalena Poplawska, Monika Ruzycka, Ireneusz P. Grudzinski, Anna M. Nowicka

**Affiliations:** 1 Faculty of Chemistry, University of Warsaw, Pasteura 1 Str., PL-02-093 Warsaw, Poland; akowalczyk@chem.uw.edu.pl; 2 Faculty of Chemistry, Warsaw University of Technology, Noakowskiego 3 Str., PL-00-664 Warsaw, Poland; akasprzak@ch.pw.edu.pl (A.K.); magdap@ch.pw.edu.pl (M.P.); 3 Faculty of Pharmacy, Medical University of Warsaw, Banacha 1 Str., PL-02-097 Warsaw, Poland; mruzycka@wum.edu.pl (M.R.); ireneusz.grudzinski@wum.edu.pl (I.P.G.)

**Keywords:** controlled drug release, cyclodextrin, lapachone, doxorubicin, polyethylenimine, cytotoxicity studies

## Abstract

This work presents a new look at the application of cyclodextrins (CD) as a drug nanocarrier. Two different cyclodextrins (*α*CD, *β*CD) were covalently conjugated to branched polyethylenimine (PEI), which was additionally functionalized with folic acid (PEI-*β*CD-*α*CD-FA). Here, we demonstrated that the combination of *α*CD and *β*CD enabled to load and control release of two anticancer drugs: doxorubicin (DOX) and beta-lapachone (beta-LP) (DOX in *β*-CD and beta-LP into *α*-CD) via host-guest inclusion. The PEI-*β*CD(DOX)-*α*CD-FA nanoconjugate was used to transport anticancer drugs into A549 lung cancer cells for estimation the cytotoxic and antitumor effect of this nanoconjugate. The presence of FA molecules should facilitate the penetration of studied nanoconjugate into the cell. Whereas, the non-cellular experiments proved that the drugs are released from the carrier mainly in the pH 4.0. The release mechanism is found to be anomalous in all studied cases.

## 1. Introduction

Cancer is a serious threat as well as one of the biggest challenges facing humanity. According to statistical data, cancer is the second most common cause of death in the world and being responsible for 9.6 million deaths in 2018 [1]. Unfortunately, despite the progress in medicine and science, the number of cancer cases is constantly growing, which is mainly explained by lifestyle and living conditions, the increase in the number of populations and, to some extent, the reduction in the incidence of other common diseases. On the one hand, the research related to the development of diagnostics and treatment of cancer diseases focuses on developing more effective methods of administering commercially available drugs to the patient, on the other, on the searching for new compounds with anticancer properties [2,3,4,5,6,7,8]. The process of searching for new cancer compounds with strong activity while maintaining low general toxicity (towards healthy tissues) and determining the mechanism of their action is long and complex. Their safe and effective use requires thorough knowledge of their pharmacological properties [9].

The effectiveness of the active ingredient of the drug in the therapy of a given disease depends, among others, on its chemical structure and applied dose. For the medicine to fulfil its role, it must reach the site of action in a timely and specific form. Standard methods of administering anti-cancer drugs do not fully use their therapeutic capabilities. Simultaneously with the cytotoxic effect on tumor cells, cytotoxic action on normal cells and tissues are observed. The reason for this is the process of distributing drugs throughout the body through the circulatory system that evenly distributes the chemotherapeutic agent in the body. As a consequence, the drug has a limited chance of reaching the target site in the therapeutic dose, i.e., to the tumor tissue without being toxic to healthy tissues. Therefore, effective drug carriers are sought that would release the active substance in a specific biological target. Polymers play key role in drug delivery technology due to facile preparation and the presence of various functional groups which can be applied in the conjugation of drug or targeting ligands, e.g., folic acid, transferrin, or peptides. One of the most commonly used polymers in this type of research is the polyethylenimine (PEI) and its derivatives [10,11,12]. The conducted research with using PEI as a drug carrier showed that such conjugates (PEI-drug) cause the reduction of the polymer toxicity, and at the same time increase in the amount of the drug delivered to the biological target [11,13,14]. It is also known that these features are strongly dependent on the molecular weight of the used polyethylenimine.

Here we showed that application of branched PEI framework allowed the covalent conjugation of *α*-cyclodextrin (*α*CD) and *β*-cyclodextrin (*β*CD). The presence of two types of supramolecular cages differing in the inner cavity size enabled loading two different anticancer drugs, namely doxorubicin (DOX) and beta-lapachone (beta-LP). Moreover, the presence of free amino groups in PEI framework provided a possibility to label such conjugate with the targeting ligand—folic acid (FA). The folate receptors are overexpressed on the surface of many of human cancers [15,16,17]. Doxorubicin is a commonly known first line therapy molecule for numerous cancers, especially including breast cancer [18,19,20], ovarian cancer [21,22,23], bladder cancer [24], or lung cancer [25,26], whereas beta-lapachone, apart from the above-mentioned cancers [27,28,29], exhibit significant antitumor activity against leukemia [30,31], prostate cancer [32], osteosarcoma [33], hepatoma [34], or pancreatic cancer [35,36,37]. We selected DOX and beta-LP for combination therapy as they act in a different manner. DOX causes DNA intercalation and disruption of topoisomerase-II-mediated DNA repair. It also generates free radicals that damage cellular membranes, DNA, and proteins [38]. In turn, beta-LP exhibits enzymatic activity of NAD(P)H:quinone oxidoreductase 1 (NQO1) that forms high levels of superoxide and peroxide damaging DNA and causing extensive NAD^+^/ATP depletion [39]. Thus, our goal herein was to introduce both DOX and beta-LP to one macromolecular nanocarrier towards combining their anticancer effects and further development of applied medicinal chemistry. In this paper we reported the procedure of the synthesis of PEI-*β*CD(DOX)-*α*CD(beta-LP)-FA nanoconjugate, as well as its full quantitative and qualitative characteristic. Additionally, the cytotoxicity of PEI-*β*CD(DOX)-*α*CD(beta-LP)-FA and its unloaded form as well as anticancer drugs (DOX and beta-LP) against adenocarcinomic human alveolar basal epithelial cells (A549) based on Alamar Blue assay were examined. We also examined pH-dependent anticancer drugs release from the nanoconjugate in the non-cellular system.

## 2. Results and Discussion

### 2.1. Characterization of Polymeric Drug Carrier

**Figure 1:**
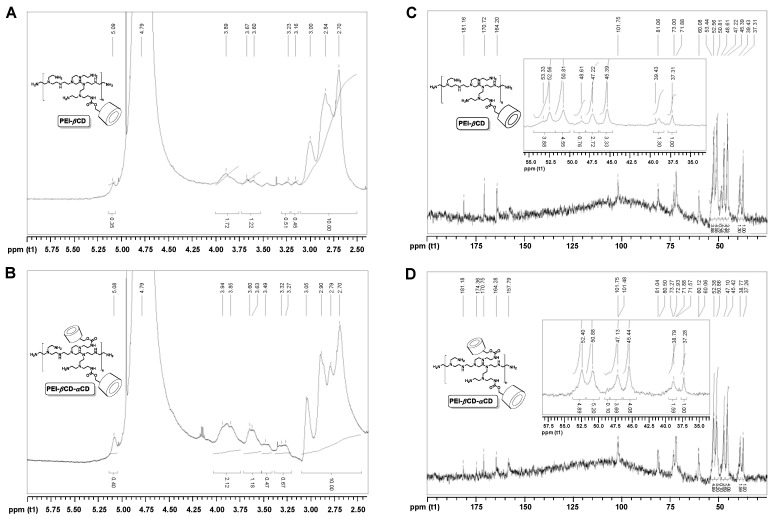
^1^H NMR spectrum (500 MHz, D_2_O) of PEI-*β*CD (**A**) and PEI-*β*CD-*α*CD (**B**). Inverse-gated ^13^C H NMR spectrum (125 MHz, D_2_O) of PEI-*β*CD (**C**). The ratio of PEI’s amino groups (NH_2_:NH:N) is 1.00:1.48:1.22, which stands for 6% of functionalization of primary amino groups of PEI with *β*CD. Inverse-gated ^13^C H NMR spectrum (125 MHz, D_2_O) of PEI-*β*CD-*α*CD (**D**). The ratio of PEI’s amino groups (NH_2_:NH:N) is 1.00:2.48:2.12, which stands for 10% of functionalization of primary amino groups of PEI-*β*CD with *α*CD.

The presence of cyclodextrins in the polymeric materials was confirmed by means of NMR spectroscopy. Conjugation of *β*CD to PEI resulted in the presence of the characteristic peaks for *β*CD (5.09 ppm, 3.89–3.60 ppm) in the ^1^H NMR spectrum of the PEI-*β*CD (Figure 1A). The broadening of the signals coming from the *β*CD unit resulted from the attachment of *β*CD to the branched PEI of high molecular weight. The peaks located at 3.23 and 3.16 ppm were ascribed to the methylene moieties between (i) carbamate linker and PEI ({*β*CD}-O-CO-NH-C**H_2_**-{PEI}) and (ii) carbamate linker and *β*CD ({*β*CD}-C**H_2_**-O-CO-NH-{PEI}), respectively. ^1^H NMR spectrum of PEI-*β*CD-*α*CD polymeric vector (Figure 1B) consisted of similar features, but the relative intensity of the CD signals with the respect to the methylene protons of PEI (3.05-2.70 ppm) increased. It clearly referred to the attachment of *α*CD units to polyethylenimine (PEI-*β*CD).

The content of CD species in the polymeric materials obtained (PEI-*β*CD and PEI-*β*CD-*α*CD) was studied with inverse-gated ^13^C NMR Spectroscopy [40,41]. The relative degree of functionalization was termed as the percent of modification of primary amino groups of PEI [42,43,44]. The amino group ratio for PEI core, that is NH_2_:NH:N, equals 1.00:1.12:0.89 for native PEI 25kDa [45]. This ratio was found to be different both for PEI-*β*CD (Figure 1C) and *α*CD-PEI-*β*CD (Figure 1D), 1.00:1.48:1.22 and 1.00:2.48:2.12, respectively. The ^13^C NMR spectra of all the polymeric products (PEI-*β*CD and PEI-*β*CD-*α*CD) consisted of the peaks characteristic for PEI and for attached CD species. What is important, the calculated content (%) of primary amino groups in PEI core decreased in the following order: PEI > PEI-*β*CD > PEI-*β*CD-*α*CD. It further supports our thesis on the success of the conjugation-type reactions between CD and PEI. From these ratios the relative degrees of modification of primary groups of PEI were calculated. It was found that the functionalization degree equaled 6% and 16%, for PEI-*β*CD (relative content of *β*CD: 6%) and PEI-*β*CD-*α*CD (relative content of *α*CD: 10%), respectively.

**Figure 2:**
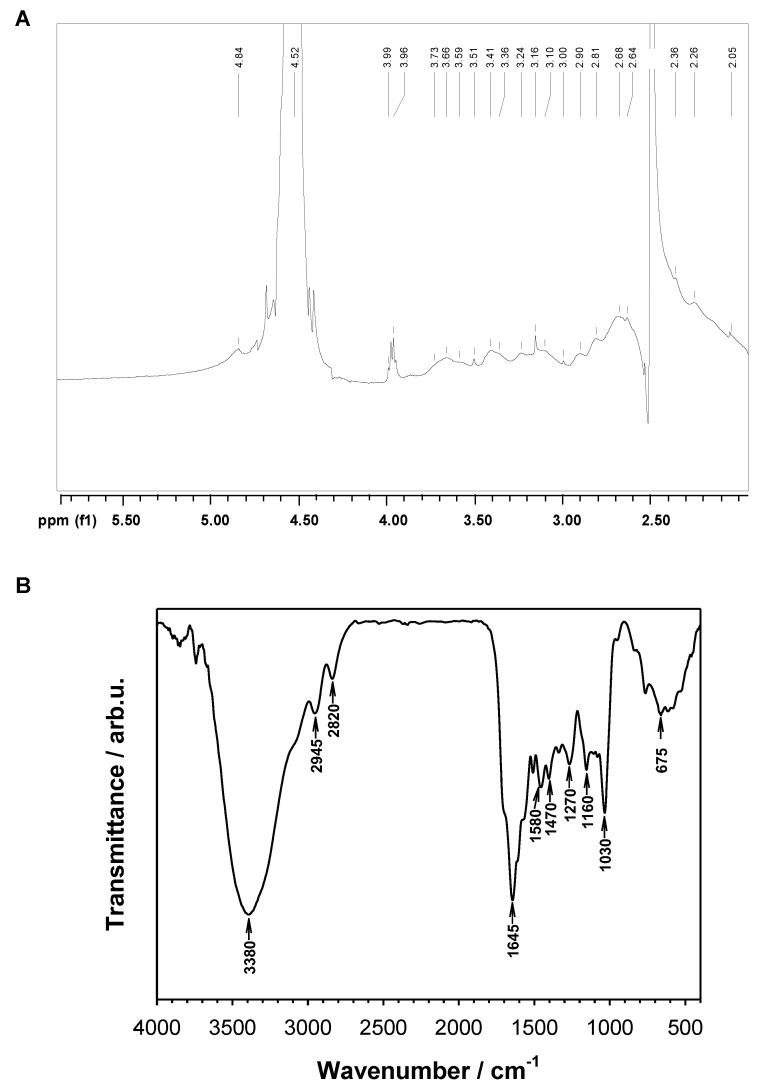
(**A**) ^1^H NMR (D_2_O/DMSO-d_6_ = 3:1 *v*/*v*, 500 MHz) spectrum of PEI-*β*CD-*α*CD-FA. (**B**) FTIR (KBr) spectrum of PEI-*β*CD-*α*CD-FA.

Successful introduction of FA to PEI-*β*CD-*α*CD material was confirmed by ^1^H NMR and FT-IR (Figure 2). Additionally, macroscopic features of the resultant material also confirmed the formation of PEI-*β*CD-*α*CD-FA; this conjugate was clearly yellow-colored, and its water solubility was significantly lower than for the parent material (PEI-*β*CD-*α*CD). The amount of introduced FA to the PEI-*β*CD-*α*CD nanoconjugate was determined from UV-vis measurements performed for 50 μM FA water solution and 0.25 mg·mL^−1^ PEI-*β*CD-*α*CD-FA. By comparison the absorbance (A) value at 359 nm the concentration of FA (6.2 μM ≡ 2.73 mg·L^−1^) in nanoconjugate was calculated according to the Lambert–Beer equation.

### 2.2. Selective Loading of Beta-LP and DOX into PEI-βCD-αCD-FA Nanoconjugate

**Figure 3:**
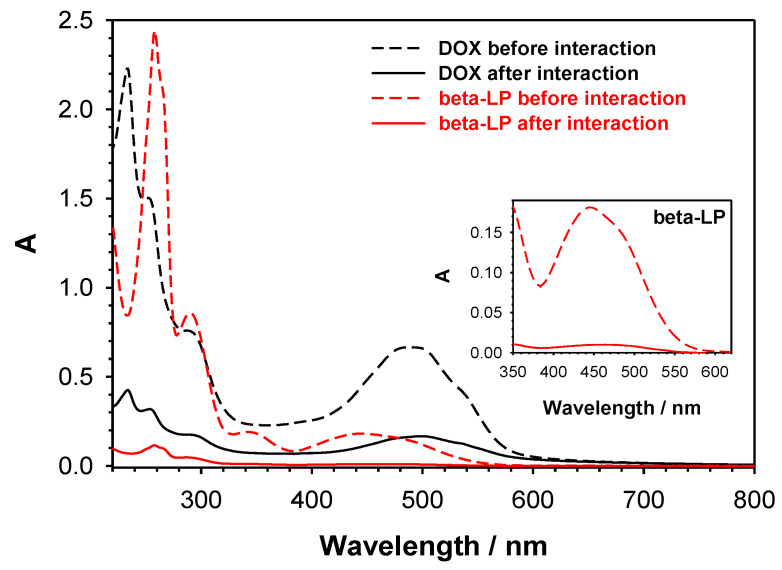
Dependences of absorbance (A) on wavelength for DOX and beta-LP solutions before and after interaction with PEI-*β*CD-*α*CD-FA nanoconjugate. Inset: Enlarged spectra for beta-LP. Experimental conditions: 0.02 M PBS (pH 7.4); $C_{\mathrm{DOX}}^{\mathrm{initial}}$ = 100 µM; $C_{\mathrm{beta}\text{-}\mathrm{LP}}^{\mathrm{initial}}$ = 100 µM.

Size-selective method for the introduction of two divergent drugs (beta-LP and DOX) to PEI-*β*CD-*α*CD-FA nanoconjugate was developed. Our idea based on the fact that the DOX molecule is too large to be encapsulated inside *α*CD cavity (pocket size 7.8 × 5.7 Å) [46,47], whilst beta-LP can fit into both *α*CD and *β*CD [48]. Thus, at first, we conducted the complexation of DOX with *β*CD unit of PEI-*β*CD-*α*CD-FA and then the remaining *α*CD residues were used to complex beta-LP. The 10-mL of water mixture containing PEI-*β*CD-*α*CD-FA nanoconjugate (0.25 mg·mL^−1^) and DOX (100 μM) was incubated overnight with stirring in ThermoMixer at the room temperature. The obtained solution was dialyzed (three times; Float-A-Lyzer^®^ G2 Dialysis Device 0.5-1K MWCO) against 0.02 M PBS buffer of pH 7.4 to remove unbound doxorubicin molecules. To determine the amount of doxorubicin loaded into *β*CD the eluate of the dialysis steps containing unbounded DOX were collected and analyzed by UV-vis spectroscopy by measuring the absorbance at ca. 490 nm. It was found that 56% (ca. 56 μM) of DOX formed an inclusion complex with βCD, whereas 44% was washed off. Next, beta-LP (100 μM) was added to the complex PEI-*β*CD(DOX)-*α*CD-FA and similarly to doxorubicin loading step, the mixture (PEI-*β*CD(DOX)-*α*CD-FA and beta-LP) was first incubated followed by a dialysis step to remove unreacted compound. The amount of unbounded beta-LP was calculated from the UV-vis spectra of eluate at ca. 359 nm and equaled ca. 5.2 μM (5.2%). Due to the small size of beta-LP molecule the interaction took place via *α*CD. Finally the obtained nanoconjugate PEI-*β*CD(DOX)-*α*CD(beta-LP)-FA was examined qualitatively and quantitatively. The UV-vis spectra of the drug solutions before and after interaction with PEI-*β*CD(DOX)-*α*CD(beta-LP)-FA nanoconjugate are presented in Figure 3.

### 2.3. Size and Stability of PEI-βCD(DOX)-αCD(beta-LP)-FA Nanoconjugate

**Figure 4:**
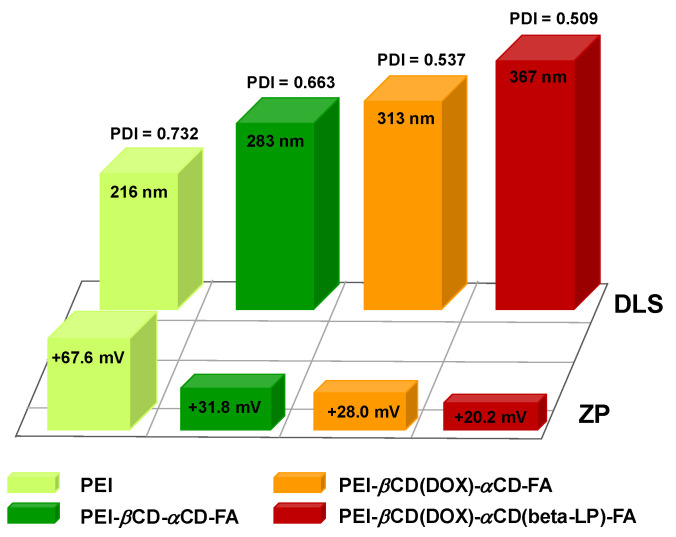
Hydrodynamic diameter values determined from DLS measurements and zeta potential values for PEI, PEI-*β*CD-*α*CD-FA nanocarrier, PEI-*β*CD(DOX)-*α*CD-FA, and PEI-*β*CD(DOX)-*α*CD(beta-LP)-FA nanoconjugates obtained in 0.02 M PBS buffer.

The main limitation of conventional chemotherapy is the small size of chemotherapeutics (doxorubicin, cis-platinum, or beta lapachon) which causes a lack of selectivity toward cancer and leads to significant accumulation in healthy organs [49]. However, carefully designed smart nanosized conjugate drug-carriers (<500 nm) could easily penetrate and accumulate inside tumor cells omitting healthy tissues mainly due to the permeability of the tumor vasculature and reduced lymphatic drainage [50]. Thus, the first stage of the study was to determine the size and stability of the carrier as well as its conjugate with DOX and beta-LP. To this end, the DLS and ZP measurements were performed. The DLS studies demonstrate that the hydrodynamic diameter of PEI-*β*CD-*α*CD-FA nanocarrier (283 nm) increases ca. 11% after loading DOX (313 nm) into αCD cavity and next after loading beta-LP into *β*CD cavity by another ca. 18% (367 nm). Moreover, the polydispersity index (PDI) decreases with subsequent PEI modifications (see Figure 4). A reduction in the PDI testifies that the size distribution of nanoparticles is narrowing. The determined nanoconjugate size indicates that it should penetrate the cells by the enhanced permeability and retention (EPR) effect. Moreover, the presence of the selective self-navigating molecule (FA) in the nanoconjugate should enhance the receptor-mediated endocytosis. To check the stability of formed PEI-*β*CD(DOX)-*α*CD(beta-LP)-FA nanoconjugate, the value of ZP was determined. The value of ZP of particles is a key indicator of the stability of a colloidal dispersion, since it reflects the ability of particles to repulse each other electrostatically [51]. The obtained ZP values, shown in Figure 4, indicate that the studied species form the stable dispersions in aqueous environment. Additionally, the loading efficiencies and stability of drugs using PEI-*β*CD-*α*CD-FA nanocarrier was promising.

### 2.4. Degradation Pathway of PEI-βCD(DOX)-αCD(beta-LP)-FA Nanoconjugate

**Figure 5:**
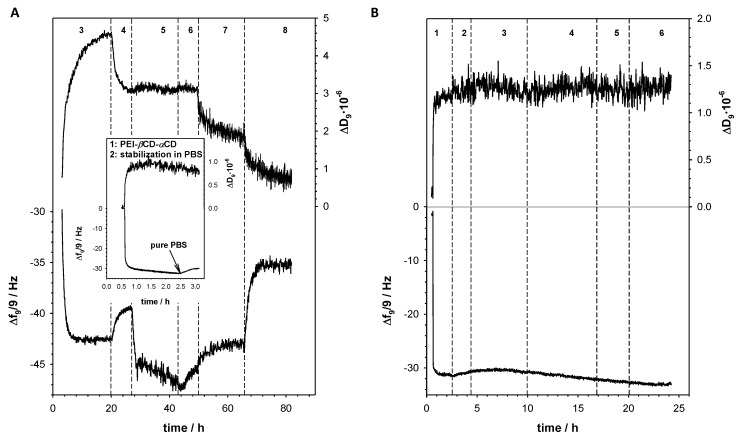
Typical QCM-D spectra of the shifts in frequency (Δ*f*) and dissipation factor (Δ*D*) recorded during subsequent steps of Au/PEI-*β*CD-*α*CD-FA (**A**) and Au/PEI (**B**) modification by DOX and beta-LP. Inset: Typical QCM-D spectra of the shifts in frequency (Δ*f*) and dissipation factor (Δ*D*) recorded during electrodeposition of PEI-*β*CD-*α*CD-FA and its stabilization. Experimental conditions: 0.02 M PBS (pH 7.4); *C*_PEI-*β*CD-*α*CD_ = 0.25 mg·mL^−1^; *C*_DOX_ = 100 µM; *C*_beta-LP_ = 100 µM. (1) Electrodeposition of PEI-*β*CD-*α*CD-FA (**A**) and PEI (**B**); (2) stabilization of PEI-*β*CD-*α*CD-FA (**A**) and PEI (**B**) layer in PBS buffer pH 7.4; (3) loading of DOX to PEI-*β*CD-*α*CD-FA (**A**) and PEI (**B**); (4) removing of unbounded DOX with PBS buffer pH 7.4; (5) loading of beta-LP to PEI-*β*CD(DOX)-*α*CD-FA (**A**) and PEI (**B**); (6) removing of unbounded beta-LP with PBS buffer pH 7.4; (7) degradation of PEI-*β*CD(DOX)-*α*CD(beta-LP)-FA at pH 5.5 (0.02 M PB buffer); (8) degradation of PEI-*β*CD(DOX)-*α*CD(beta-LP)-FA at pH 4.0 (0.02 M PB buffer).

Due to the fact of application of FA (2.73 mg FA per 1 g PEI-*β*CD-*α*CD-FA) as targeting ligand, the internalization into the cell through receptor-mediated endocytosis is favored. Depending on the macromolecule structure and kind of endocytosis process involved, a series of sorting steps take place in which the endosome is either transported to the certain cell organelles, returns to the cell surface, or forms primary and secondary lysosomes. Circulation of the endosome inside the cell is closely related to the changes in pH from pH 7.2–7.4 in the extracellular space, to pH 6.5–5.0 in the endosomes and around pH 4.5–4.0 in primary and secondary lysosomes [52]. Therefore, the conjugate drug-carrier can be degraded in the cancer cell by two major fundamentally different mechanisms lysosomotropic and/or endosomotropic pathway, in which the drug is released from the lysosome or endosome to the cytoplasm, respectively [53,54]. To determine the drug release from the PEI-*β*CD(DOX)-*α*CD(beta-LP)-FA nanoconjugate, the QCM-D experiments were carried out in the solutions with various values of pH: 7.4, 5.5, 4.0 using the 3rd to 13th overtones. Figure 5A shows the frequency (Δ*f*) and the dissipation factor (Δ*D*) changes during formation the inclusion complex of DOX with *β*CD and beta-LP with *α*CD and their releasing in various values of pH. Before the experiments the gold quartz crystal was modified with PEI-*β*CD-*α*CD-FA nanoconjugate through electrodeposition at constant potential (*C*_PEI-*β*CD-*α*CD-FA_ = 0.25 mg·mL^−1^ in 0.5 M NaCl; −0.05 V, t = 7200 s). Next, the obtained layer was stabilized by recording continuous cyclic voltammograms in PBS buffer, in potential range from 0 to 0.7 V, with scan rate 100 mV·s^−1^, until a stable voltammogram was observed. The mass of PEI-*β*CD-*α*CD-FA nanoconjugate introduced onto the quartz crystal surface was 0.522 μg·cm^−2^ (*m*_PEI-*β*CD-*α*CD-FA_ = −Δ*f*·*C*_f_ 29.5 Hz·17.7·ng·cm^−2^, where *C*_f_ = 17.7 ng·cm^−2^ is the mass sensitivity of the applied QCMD). After stabilization of the frequency of the modified electrode (Au/PEI-*β*CD-*α*CD-FA) in 0.02 M PBS buffer at pH 7.4, the doxorubicin solution (100 μM) was added to the reaction chamber and the drop in Δ*f* value was observed. This decrease was a consequence of formation of inclusion complex between *β*CD and DOX. After ca. 8 h the frequency shift reached a stable value −42.5 Hz. Then the solution in the chamber was changed on the pure 0.02 M PBS buffer (pH 7.4) to remove unbounded DOX molecules; during this step the frequency increased to the value −39.5 Hz. The apparent mass increase relating to the observed experimental frequency shift (−Δ*f* = 10 Hz) for DOX loading in *β*CD supramolecular cages is *m*_DOX_ = 10·17.7 = 177.0 ng·cm^−2^. After stabilization of the frequency and dissipation valuesthe beta-LP solution (100 μM) was introduced to the reaction chamber and the drop in Δ*f* value was again observed. Similarly to the case of doxorubicin, after the stabilization of the frequency an elution step of unreacted beta-LP using a PBS buffer took place. The apparent mass increase relating to the observed experimental frequency shift (−Δ*f* = 6 Hz) for beta-LP loading in *α*CD molecular cages is *m*_beta-LP_ = 6·17.7 = 106.2 ng·cm^−2^. To get the information about releasing of DOX and beta-LP from the PEI-*β*CD(DOX)-*α*CD(beta-LP)-FA nanoconjugate, phosphate buffer of two different pH values (5.5 and 4.0) was applied. The application of PBS buffer of pH 5.5 led to the increase of the frequency of the quartz crystal to the value −43 Hz and a decrease of the dissipation factor. In turn, the use of a lower pH buffer led to a further increase in frequency up to the value −35 Hz.

To prove that the drugs are introduced to the nanoconjugate only through formation inclusion complexes with CD, the control experiment with unmodified PEI was performed using QCM-D. Before the measurements, gold quartz crystal was modified with PEI through electrodeposition at constant potential (C_PEI_ = 0.25 mg·m·L^−1^ in 0.5 M NaCl; −0.05 V, t = 7200 s). As it is seen in Figure 5B, no frequency and dissipation factor changes were observed during the interaction of DOX and beta-LP with PEI. It means that inclusion was only one way of introducing the drugs into the PEI-βCD-αCD-FA nanoconjugate.

**Figure 6:**
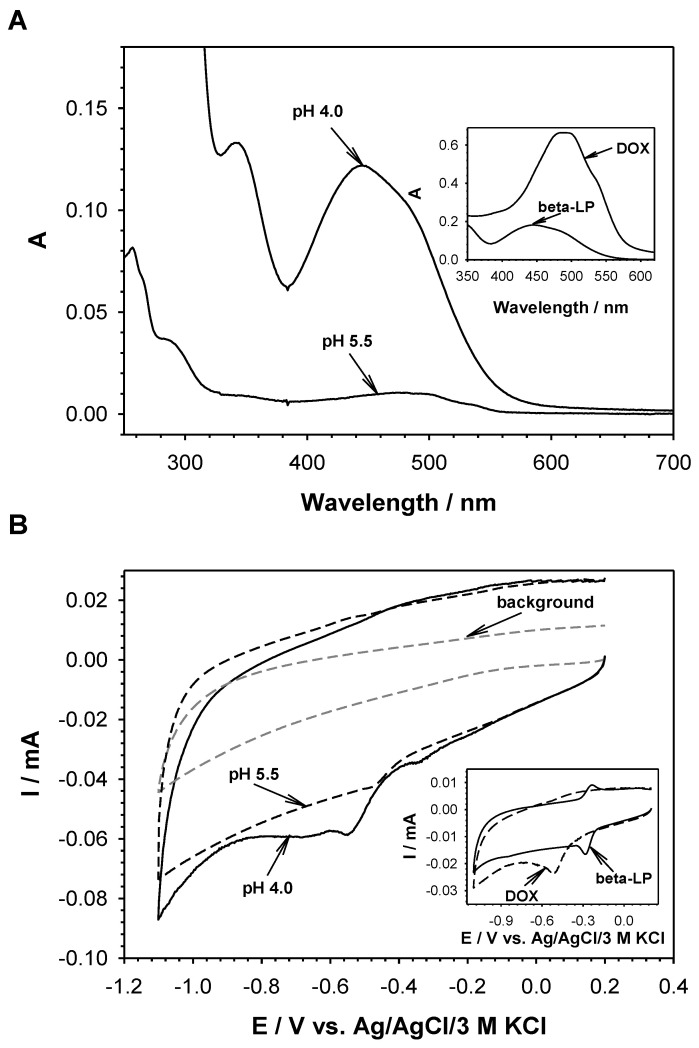
UV-vis spectra (**A**) and cyclic voltammograms (**B**) of the eluates obtained at pH 5.5 and 4.0. Insets: UV-vis spectra (**A**) and cyclic voltammograms (**B**) of DOX and beta-LP. Inset A: UV-vis spectra of 100 µM solutions of pure DOX and beta-LP. Inset B: Cyclic voltammograms of 100 µM solutions of pure DOX and beta-LP (scan rate: 100 mV·s^−1^; glassy carbon disc electrode *φ* = 3 mm).

To determine what drug, and under what conditions, is released from the conjugate, the UV-vis and voltammetric measurements of the obtained eluates were performed (Figure 6). The analysis of collected eluates showed that at pH 5.5 only a part of complexed DOX was released from the nanoconjugate, whereas at pH 4.0 both DOX and beta-LP were released from the complexes with cyclodextrins. It means that the degradation of PEI-*β*CD(DOX)-*α*CD(beta-LP)-FA could plausibly occur at pH 4.0.

**Figure 7:**
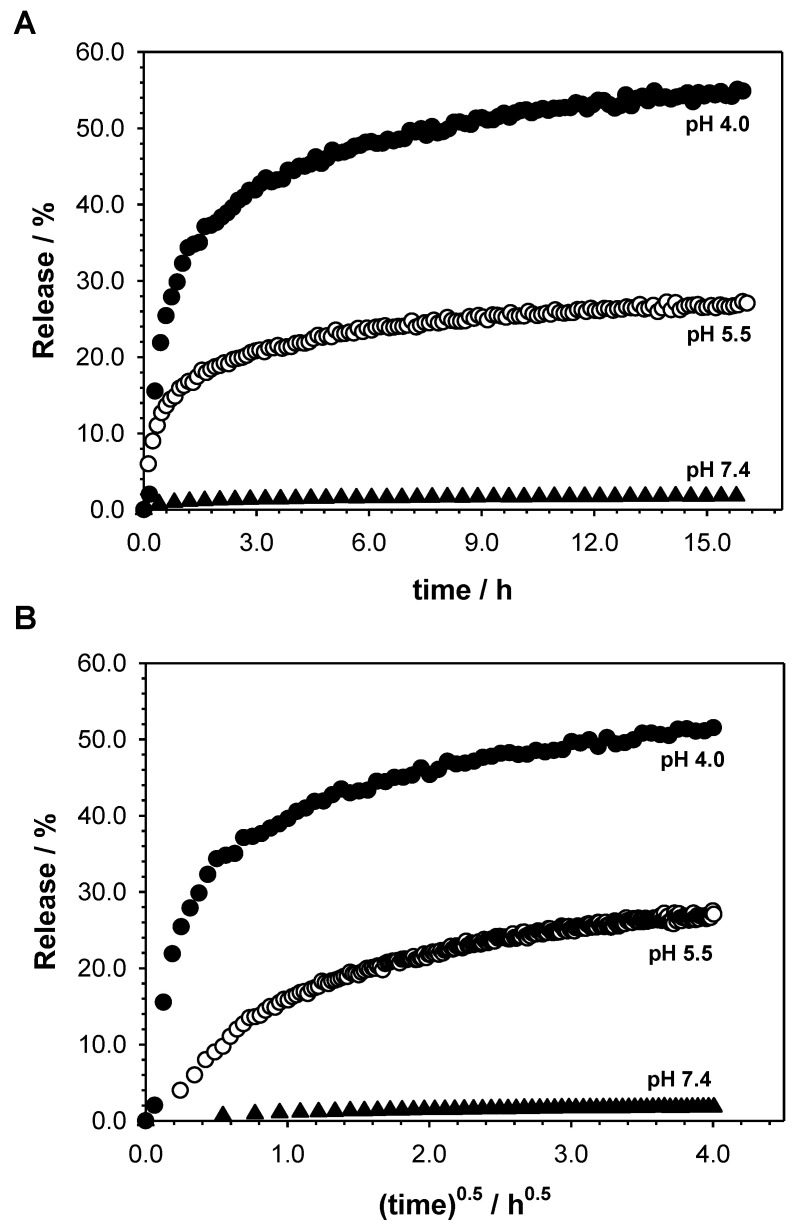
The in vitro release profiles of DOX and beta-Lp from PEI-*β*CD(DOX)-*α*CD(beta-LP)-FA nanoconjugate in two media according to Korsmeyer–Peppas (**A**) and Higuchi (**B**) models.

Kinetics of the drug release process from PEI-*β*CD(DOX)-*α*CD(beta-LP)-FA nanoconjugates were assessed based on the amount of released drugs into the buffer at pH 5.5 and 4.0. The release profiles and release parameters are shown in Figure 7 and Table 1, respectively. The release mechanism of drugs is found to be anomalous in all studied cases. It should be stressed that the release constant rate (k_H_) was higher in the pH 4.0 than in the pH 5.5. Under the experimental conditions, the relatively slow release of drugs from the PEI-*β*CD(DOX)-*α*CD(beta-LP)-FA nanoconjugate was achieved. This is desirable for the development of new biomaterials as drug carriers because they provide therapeutic concentrations of the drug substance at the implantation site for a longer period of time [55]. None of the tested drugs were 100% released from the carrier. It is worth stressing that the high rate of drug release at acidic pH is beneficial towards prospective biological applications of our nanoconjugate, since it is well-known that cancer cells’ environments show acidic pH values (pH 4–6) [56,57]. Additionally, no significant drugs release at pH 7.4 suggests that one can expect controlled drug release at the therapeutic target (cancer cells) and no uncontrolled drug release in bloodstream (cell targeting stage).

### 2.5. Biological Screening

The important objective of this study was to determine the cytotoxic effect of the as-obtained compounds loaded with doxorubicin and beta-lapachone. For data, the Alamar Blue assay was used. Note that the Alamar Blue assay enables establishment of the relative cytotoxicity of agents within various chemical classes including nanoparticles by quantitative measurement of the proliferation of various human and animal cell lines due to the fluorescent indicator dye resazurin. It is a blue, non-fluorescent, and cell permeable oxidation-reduction indicator, which upon multiple metabolic reactions occurring in living cells are reduced to red and highly fluorescent resorufin. The absorbance- and fluorescence-based measurement of the redox indicator proportionally reflects the number of living and metabolic active cells [58]. Toxicants like nanoparticles may disrupt the metabolism of living cells by triggering cell death and/or inhibition of cell growth. Such modification in number of living cells expresses the cytotoxicity of the toxic agent.

**Figure 8:**
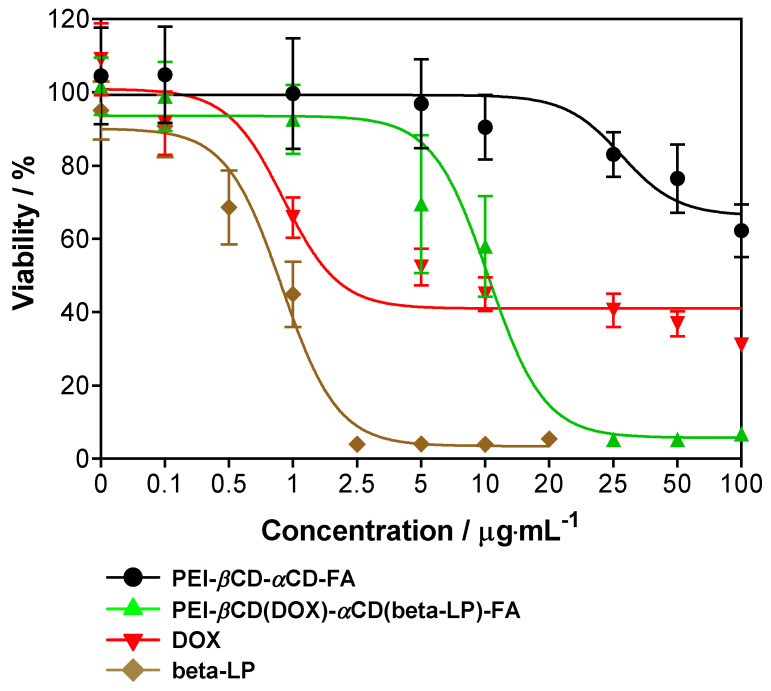
The viability of A549 cells treated with nanoconjugate transporters loaded with and without doxorubicin and beta-lapachone and free doxorubicin and beta-lapachone for 24 h. Data are mean ± SD from three independent experiments.

In the present study, A549 cells were treated with PEI-*β*CD-*α*CD-FA, PEI-*β*CD(DOX)-*α*CD(beta-LP)-FA, free DOX and free beta-lapachone. After 24 h of incubation, over 90% inhibition of the growth of A549 cells was observed due to the treatment at 2.5 µg·mL^−1^ of beta-lapachone (Figure 8). Note that ca. 10 fold higher dose of PEI-*β*CD(DOX)-*α*CD(beta-LP)-FA was needed to reach the same inhibitory effect (Figure 8). The lowest cytotoxicity was observed for the PEI-*β*CD-*α*CD-FA sample (Figure 8). The calculated *IC*_50_ values for DOX (*IC*_50_ = 0.78 µg·mL^−1^) and beta-lapachone (*IC*_50_ = 0.81 µg·mL^−1^) tested alone were found to be ca. 10-fold lower as compared to those calculated for the PEI-*β*CD(DOX)-*α*CD(beta-LP)-FA construct loaded with these agents (*IC*_50_ = 8.54 µg·mL^−1^). Note that the PEI-*β*CD(DOX)-*α*CD(beta-LP)-FA construct at the *IC*_50_ (*IC*_50_ = 8.54 µg·mL^−1^) value contains 1.09 µg·mL^−1^ of DOX and 0.79 µg·mL^−1^ of beta-lapachone. As only 60% of these agents are expected to be liberated from the PEI-*β*CD(DOX)-*α*CD(beta-LP)-FA construct as estimated from in vitro drug release studies (Figure 7), therefore, we assumed that respective 0.66 and 0.47 µg·mL^−1^ of DOX and beta-lapachone are finally revealed after introduction into the A549 cells. Treating the cells with the uploaded PEI-*β*CD(DOX)-*α*CD(beta-LP)-FA sample at the *IC*_50_ level, it seems that the final DOX and beta-lapachone concentrations are expected to be ca. 20% and ca. 40% lower than *IC*_50_ of free DOX and *IC*_50_ of free beta-lapachone, respectively. On the other hand, simultaneous application of free doxorubicin and free beta-lapachone could give information about their anticancer efficacy at *IC*_50_ concentrations. Nevertheless, higher concentrations of both anticancer agents do not have to stand for greater anticancer activity. Note that the place of action of both DOX and beta-LP is at DNA level, meaning that they need to reach a cell’s interior that can be facilitated exactly due to FA incorporation into nanosystem construction. Definitely, further in vitro and in vivo studies need to be done in future in order to estimate other pharmacological and toxicological effects of this construct in A549.

## 3. Materials and Methods

### 3.1. Materials

Branched polyethylenimine (>99%; MW (by LS): 25 kDa), *β*-cyclodextrin (>97%), *α*-cyclodextrin (> 98%), beta-lapachone (beta-LP; ≥98%), doxorubicin (DOX; ≥98%), folic acid (FA; ≥97%), *N*-hydroxysuccinimide (NHS, 98%), and *N*-(3-dimethylaminopropyl)-*N*′-ethylcarbodiimide hydrochloride (EDCl; ≥99%) were purchased from Sigma-Aldrich, Poznań, Poland. 1,1′-Carbonyldiimidazole (98%) was purchased from Fluorochem. Triethylamine (>99.8%) and dimethylsulfoxide (DMSO; >99%) were purchased from Avantor Performance, Gliwice, Poland, whereas NaH_2_PO_4_, Na_2_HPO_4_, NaCl, KCl were purchased from POCH, Gliwice, Poland. All reagents were used as received without further purification. All non-cellular studies were performed in 0.02 M phosphate buffer with addition of 2 mM KCl and 150 mM NaCl (PBS), at various pH (7.4, 5.5, and 4.0).

### 3.2. Synthesis of PEI-βCD-αCD-FA Nanoconjugate and its Complex with DOX and Beta-LP

**Scheme 1:**
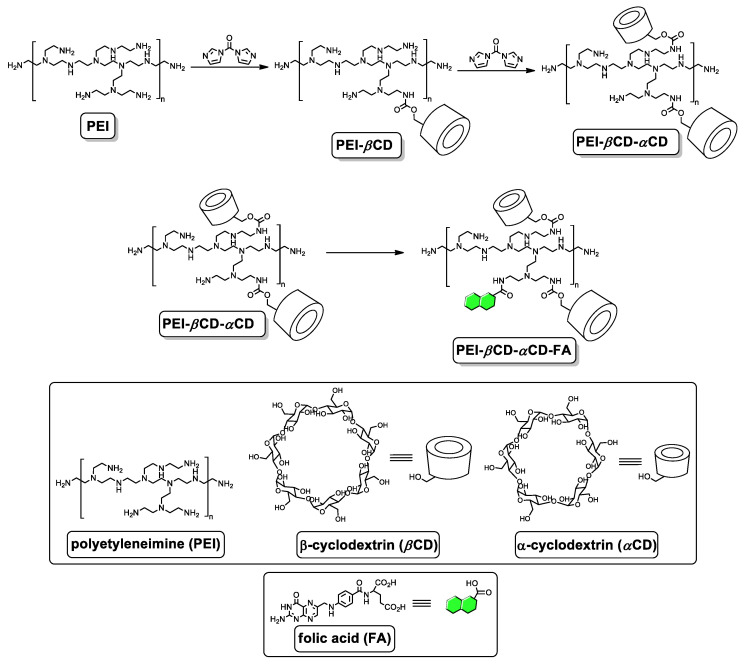
Synthesis of PEI-*β*CD, PEI-*β*CD-*α*CD, and PEI-*β*CD-*α*CD-FA. Folic acid molecules are marked in green.

Cyclodextrin species, namely *α*CD and *β*CD, were conjugated to branched PEI of molecular weight (M_n_) 25 kDa by means of stepwise 1,1′-carbonyldiimidazole (CDI)-mediated reactions (Scheme 1). Briefly, a DMSO solution containing *β*CD activated with CDI was added to the aqueous solution of PEI 25k, and after the completion of the reaction, the polymeric material was purified by means of dialysis against water (3.5k MWCO membrane was used). The as-obtained polymeric material (PEI-*β*CD) was subjected to the reaction with *α*CD activated with CDI. Finally, after dialysis against water (3.5K MWCO Snake-Skin^®^ Dialysis Tubes, Thermo Fisher Scientific, Warsaw, Poland) the desired polymeric vector (PEI-*β*CD*-α*CD) was obtained. The lyophilization was performed using FreeZone 1 L Laboratory Lyophilizer (LABCONCO, Gadńsk, Poland). The details of synthesis of individual components of the nanoconjugate are given below.

### 3.3. Synthesis of PEI-βCD

The flask containing *β*CD (170.4 mg, 0.15 mmol) was purged with argon and a DMSO (4 mL) solution containing CDI (194.4, 1.20 mmol) and triethylamine (TEA; 90 μL, 121.4 mg, 1.20 mmol). The mixture was then stirred at room temperature for 3 h under argon atmosphere. The solution was added to an aqueous solution (10 mL) of PEI (500 mg). The reaction mixture was stirred for 24 h at room temperature. After dialysis against distilled water for 5 days (3.5k MWCO membrane) and lyophilization for 24 h, 392.5 mg of PEI-*β*CD was obtained.

^1^H NMR (D_2_O, 500 MHz, ppm): δ_H_ 5.09–5.08 (bm, *β*CD), 3.89–3.80 (bm, *β*CD), 3.67–3.60 (bm, *β*CD), 3.23–3.22 (bm, {*β*CD}-O-CO-NH-C**H_2_**-{PEI}), 3.16–3.15 (bm, {*β*CD}-C**H_2_**-O-CO-NH-{PEI}), 3.00–2.70 (bm, PEI).

^13^C NMR (D_2_O, 125 MHz, ppm): δ_C_ 181.16, 170.72, 164.20, 101.75, 81.06, 73.00, 71.88, 60.08, 53.44, 52.56, 50.81, 48.61, 47.22, 45.39, 39.43, 37.31.

Ratio of amino groups in the polymeric material (NH_2_:NH:N): 1.00:1.48:1.22.

### 3.4. Synthesis of PEI-βCD-αCD

The flask containing *α*CD (58.4 mg, 0.06 mmol) was purged with argon and a DMSO (2 mL) solution containing CDI (77.8, 0.48 mmol) and TEA (35 μL, 48.6 mg, 0.48 mmol). The mixture was then stirred at room temperature for 3 h under argon atmosphere. The solution was added to an aqueous solution (4 mL) of PEI-*β*CD (200 mg). The reaction mixture was stirred for 24 h at room temperature. After dialysis against distilled water for 5 days (3.5k MWCO membrane) and lyophilization for 24 h, 392.5 mg of PEI-*β*CD-*α*CD was obtained.

^1^H NMR (D_2_O, 500 MHz, ppm): δ_H_ 5.08–5.07 (bm, *β*CD and *α*CD), 3.94–3.85 (bm, *β*CD and *α*CD), 3.65–3.63 (bm, *β*CD and *α*CD), 3.49–3.47 (bm, {*β*CD}-O-CO-NH-C**H_2_**-{PEI} and {*α*CD}-O-CO-NH-C**H_2_**-{PEI}), 3.32–3.27 (bm, {*β*CD}-C**H_2_**-O-CO-NH-{PEI} and {*α*CD}-C**H_2_**-O-CO-NH-{PEI}), 3.05-2.70 (bm, PEI).

^13^C NMR (D_2_O, 125 MHz, ppm): δ_C_ 181.18, 174.36, 170.75, 164.28, 157.79, 101.75, 101.48, 81.04, 80.50, 73.27, 72.93, 71.88, 71.57, 60.12, 60.06, 52.38, 50.86, 47.10, 45.42, 38.77, 37.26.

Ratio of amino groups in the polymeric material (NH_2_:NH:N): 1.00:2.48:2.12.

### 3.5. Synthesis of PEI-βCD-αCD-FA

Prior to the conjugation, FA (33 mg, 0.075 mmol) was activated with EDC (28.8 mg, 0.15 mmol) and NHS (17.3 mg, 0.15 mmol) for 2 h in DMSO (2 mL) at room temperature under argon atmosphere. The solution of activated FA was added to a stirred solution of *α*CD-PEI-*β*CD (150 mg) in distilled water (10 mL). The reaction mixture was stirred for 24 h at room temperature. After dialysis against distilled water for 7 days (3.5k MWCO membrane) and lyophilization for 24 h, 172.1 mg of PEI-*β*CD-*α*CD-FA was obtained. Note: due to too low solubility of this derivative in deuterated solvents, the analyses were based on ^1^H NMR (Figure 2A; D_2_O/DMSO-d_6_ = 3:1 v/v solvent system) and FT-IR spectra (Figure 2B), the acquisition of ^13^C NMR spectrum was not possible despite the measurement for more than 50 h.

^1^H NMR (D_2_O/DMSO-d_6_ = 3:1 *v*/*v*, 500 MHz, ppm): δ_H_ 4.84–4.83 (bm), 3.99–3.96 (bm), 3.73–3.59 (bm), 3.51 (bs), 3.41–3.36 (bm), 3.24–3.16 (bm), 3.10 (bs), 2.90–2.81 (bm), 2.68–2.64 (bm), 2.46 (bs), 2.26 (bs), 2.05 (bs). FT-IR (KBr, cm^−1^) *v* 3380, 2945, 2820, 1645, 1580, 1470, 1270, 1160, 1030, 675.

### 3.6. Cell Culture

The A549 (ATCC^®^ CCL-185™) cell line was obtained from American Type Culture Collection (ATCC, Manassas, VA, USA). The culture was cultivated in a 5% CO_2_ atmosphere at 37 °C in CO_2_ incubator (Memmert, Schwabach, Germany). The cells were grown as adherent monolayers in F-12K Medium (Kaighn’s Modification of Ham’s F-12 Medium, Gibco, Paisley, UK), supplemented with 10% FBS (Fetal Bovine Serum, Gibco, Paisley, UK) and antibiotics: streptomycin, 50 μg·mL^−1^; amphotericin B, 1.25 μg·mL^−1^; gentamicin 50 μg·mL^−1^; penicillin, 50 U·mL^−1^ (Gibco, Paisley, UK).

### 3.7. Biological Screening

The cytotoxicity assessment was performed based on Alamar Blue assay, which was carried out according to a manufacturer’s instruction (Alamar BlueTM Cell Viability Reagent, ThermoFisher Scientific, Life Technologies Corporation, Eugene, Oregon, USA). A549 cells were trypsinized (0.25% trypsin/EDTA solution, Gibco, Paisley, UK) and plated in 96-well plates (Falcon, Corning, Durham, USA) at a density of 105 cells/mL per well. Then, 24 h after cell adhesion, A549 cells were exposed to PEI-*β*CD-*α*CD-FA and PEI-*β*CD(DOX)-*α*CD(beta-LP)-FA at various concentrations, which were further incubated for 24 h. Anticancer drug doxorubicin (DOX) and beta-lapachone (beta-LP) served as reference compounds. Control cells were incubated with media. After incubation, the control medium and investigated compounds were removed, then the cells were rinsed twice with PBS and 100 μL of Alamar Blue solution (10% [*v*/*v*] solution of Alamar Blue dye in fresh medium) was transferred to each well. Following 3 h of incubation (37 °C, 5% CO_2_, 90% humidity), the Alamar Blue fluorescence was quantified at the excitation and emission wavelengths of 570 and 600 nm using an Epoch microplate reader (BioTek). The percent viability was expressed as fluorescence counts in the presence of test compound as a percentage of that in the control.

### 3.8. Applied Characterization Techniques

All NMR experiments were carried out on a Varian VNMRS spectrometer operating at 500 MHz (Varian Inc., Yarnton, UK) and equipped with a multinuclear z-gradient inverse probehead. The temperatures of the probes were maintained at 298 K and standard 5 mm NMR tubes were used. ^1^H NMR and inverse-gated ^13^C NMR spectra were recorded in deuterium oxide (with the calibration on the residual HOD signal 4.79 ppm and the *β*-cyclodextrin’s C1 signal (*β*CD −101.75 ppm). No internal reference was added, in order to eliminate possible interactions with *β*CD, if any. MestRe-C 2.0 software was used for NMR spectra simulation (MestRe-C NMR Data Processing Made Easy 4.9.9.6, 1996–2006, courtesy F.J. Sardina, Universidad de Santiago de Compostela, Spain). Fourier-transform infrared (FT-IR) spectra were recorded in the transmission mode in a form of pellets with KBr with the Thermo Nicolet Avatar 370 spectrometer with spectral resolution of 4 cm^−1^ (100 scans).

QCM-D studies were carried out with a QEM 401 instrument with the QSoft software (Q-Sense, Biolin Scientific, Darmstadt, Germany) equipped with 4.95 MHz AT-cut gold-coated quartz crystals. Before each experiment the Au crystals were cleaned by immersing them, for 5 min, in the heated (75 °C) mixture of ultrapure water, 25% ammonia, and 30% hydrogen peroxide in the volume ratio 5:1:1 to oxidize all unwanted species attached to the gold surface. Next, the crystals were rinsed with ultrapure water and dried with Ar stream. The measurements were performed in the flow system with the flow rate of 100 µL·min^−1^ and constant temperature 21 °C.

Cyclic voltammetry was performed using an Autolab, model PGSTAT 12 potentiostat equipped with an ECD amplifier module (RC time settings: 0 s for scan rates > 10 mV/s and 0.1 s for scan rates < 10 mV/s; where RC is a filter time constant that helps to filter out noise), and the electrochemical analysis system based on the GPES software package (Eco Chemie B. V., Utrecht, Netherlands). The experiments were carried outin the three electrode system consisting of a glassy carbon disc electrode (*φ* = 3 mm, BASi, West Lafayette, IN, USA) as working electrode, Ag/AgCl/3 M KCl as a reference electrode, and platinum plate as a counter electrode. The electrode surface was cleaned before each experiment on the wet pad with Al_2_O_3_ (*φ* = 1 µM). After polishing the electrode surface was extensively washed with ultrapure water (Hydrolab, Gdańsk, Poland; conductivity of ≈0.056 µS·cm^−1^) to remove alumina from its surface.

UV-vis spectra were recorded using PerkinElmer spectrometer Lambda 25 (Waltham, MA, USA), at room temperature in quartz cuvette of 1 cm length of optical window.

The dynamic light scattering (DLS) and zeta potential (ZP) measurements were performed with a Zetasizer nano series apparatus (Malvern Panalytical Ltd., Malvern, UK) with a He-Ne (4 mW) laser at 632.8 nm. The experiments were carried out in 0.02 M PBS buffer at 21 °C, at least five times, with two freshly prepared samples.

### 3.9. Kinetic Analysis of Drug Release In Vitro Profiles

The drug releasing studies (QCM-D) were performed in PBS buffer at various pH (7.4, 5.5, and 4.0) in the non-cellular system. Drug release kinetics were adjusted to selected theoretical models, i.e., Higuchi and Korsmeyer–Peppas, using Equations (1) and (2) describing these models, respectively. Data obtained from release studies were fitted to equation:

*Q* = *k*_H_*t*^0.5^ (1)

where *Q* is the percentage of drug release at time *t*, *k*_H_ is a release rate constant (Higuchi constant).

*Q* = *kt^n^* (2)

where *Q* is the percentage of drug release at time *t*, *k* is a release rate constant, and *n* is release exponent. The *n* value is used to characterize different release mechanism [59]. According to Korsmeyer–Peppas model for *n* ≤ 0.43 drug release kinetics occur according to Fickian diffusion, for 0.43 < *n* < 0.85 the release is described as anomalous and for *n* ≥ 0.85 drug release is dominated by an erosion mechanism.

## 4. Conclusions

In this work, the newly synthesized nanoconjugate composed of branched PEI covalently conjugated to *α*-cyclodextrin, *β*-cyclodextrin, and functionalized with folic acid was applied as a drug nanocarrier for doxorubicin and beta-lapachone. The drugs were loaded into supramolecular cages via formation of inclusion complexes. The effectiveness of the covalent conjugation of cyclodextrins and folic acid was confirmed by FTIR, UV-vis, and NMR analysis. The presence of folic acid as a targeting ligand facilitating specific binding to folate receptors (FR) on the surface of various cancerous cells provide an efficient FR-mediated endocytosis and targeted delivery of drug to tumor tissue.

Under physiological conditions, pH 7.4, the PEI-*β*CD(DOX)-*α*CD(beta-LP)-FA nanoconjugate is stable, as elucidated by QCM-D studies, as well as dynamic scattering and zeta potential assays. The degradation of PEI-*β*CD(DOX)-*α*CD(beta-LP)-FA occurs in the acidic conditions. Despite the fact that the drugs were introduced to the nanocarrier via weak interactions (inclusion complex), only 60% of loaded drugs were released, of which about 20% during pH 5.5, and the remaining part at pH 4.0. The UV-vis and voltammetric analyses of eluates, obtained at different pH environments, proved that the doxorubicin is much more easily liberated from the nanoconjugate than beta-LP. Incomplete drugs liberation was also observed in cytotoxicity screening. Although the nanosystem construction allows drug attachments at effective amounts, 60% release of DOX and beta-lapachone from the loaded PEI-*β*CD(DOX)-*α*CD(beta-LP)-FA nanocarrier results in lower drug concentrations than calculated *IC*_50_ of their free forms. If this nanocarrier was able to release 100% of the loaded drugs, then DOX could be delivered at the concentration higher than *IC*_50_ and beta-lapachone at the concentration almost equal to *IC*_50_ of their free forms plausibly resulting in a more satisfying cytotoxicity effect in the studied cells. Therefore, further improvements need to be done especially in context of the controlled drug release from the as-designed nanocarrier. Nevertheless, the proposed nanocarrier still has a great potential to serve as a nanotransporter targeting the anticancer cargo directly to the cancer cells due to folate ligands. Note that DOX and beta-LP delivery into cancer cells through the FA decorated nanoconstruct may significantly decrease overall systemic cytotoxicity of these agents. Therefore, this innovative concept of simultaneous loading of different chemical agents in one nanoconstruct can be valuable groundwork for further development of more effective and sophisticated nanoplatforms.

## Figures and Tables

**Table 1:** Change in coefficient (*r*), release rate constant (*k*_H_), and release exponent (*n*) for studied PEI-*β*CD(DOX)-*α*CD(beta-LP)-FA nanoconjugate.

Media	Higuchi		Korsmeyer–Peppas	
	*k*_H_ [μg/h^−0.5^]	*r* ^2^	*n*	*r* ^2^
PEI-*β*CD(DOX)-*α*CD(beta-LP)-FA				
pH 5.5	16.9	0.966	0.471	0.968
pH 4.0	56.3	0.983	0.720	0.928

## Abbreviations

beta-LP	beta-lapachone
CD	cyclodextrins
DLS	dynamic light scattering
DOX	doxorubicin
FA	folic acid
FR	folate receptor
FTIR	Fourier-transform infrared spectroscopy
*IC*_50_	half maximal inhibitory concentration
NMR	nuclear magnetic resonance spectroscopy
PBS	phosphate-buffered saline
PEI	polyethylenimine
PEI-*α*CD	nanoconjugate of polyethylenimine with alpfa cyclodextrins
PEI-*β*CD	nanoconjugate of polyethylenimine with beta cyclodextrins
PEI-*β*CD-*α*CD-FA	nanoconjugate of polyethylenimine with alpha and beta cyclodextrins and folic acid
PEI-*β*CD(DOX)-*α*CD(beta-LP)-FA	nanoconjugate of polyethylenimine with alpha and beta cyclodextrins and folic acid loaded with doxorubicin and beta-lapachone
QCM-D	quartz crystal microbalance with dissipation monitoring
ZP	zeta potential

## References

[1] Bray F., Ferlay J., Soerjomataram I., Siegel R.L., Torre L.A., Jemal A. (2018). Global cancer statistics 2018: GLOBOCAN estimates of incidence and mortality worldwide for 36 cancers in 185 countries. CA A Cancer J. Clin..

[2] Divekar K., Swamy S., Murugan V. (2020). Synthesis and evaluation of some newer pyrazolines as possible potential antitumor agents. Int. J. Pharm. Sci. Res..

[3] Kuo Y.-S., Zheng M.-Y., Huang M.-F., Miao C.-C., Yang L.-H., Huang T.-W., Chou Y.-T. (2020). Association of Divergent Carcinoembryonic Antigen Patterns and Lung Cancer Progression. Sci. Rep..

[4] Leal A.I.C., Van Grieken N.C.T., Palsgrove D.N., Phallen J., Medina J.E., Hruban C., Broeckaert M.A.M., Anagnostou V., Adleff V., Bruhm D.C. (2020). White blood cell and cell-free DNA analyses for detection of residual disease in gastric cancer. Nat. Commun..

[5] Liu Y., Wu Y., Sun L., Gu Y., Hu L. (2020). Synthesis and structure-activity relationship study of water-soluble carbazole sulfonamide derivatives as new anticancer agents. Eur. J. Med. Chem..

[6] Reyna M.A., Haan D., Paczkowska M., Verbeke L.P.C., Vazquez M., Kahraman A., Pulido-Tamayo S., Barenboim J., Wadi L., Dhingra P. (2020). Pathway and network analysis of more than 2500 whole cancer genomes. Nat. Commun..

[7] Saraiva N., Costa J.G., Reis C.P., Almeida N., Rijo P., Fernandes A.S. (2020). Anti-Migratory and Pro-Apoptotic Properties of Parvifloron D on Triple-Negative Breast Cancer Cells. Biomolecules.

[8] Zhang S., Wang D., Huang J., Hu Y., Xu Y. (2019). Application of capsaicin as a potential new therapeutic drug in human cancers. J. Clin. Pharm. Ther..

[9] Brunton L.L., Lazo J.S., Parker K., O Buxton I.L., Blumenthal D., Csákó G. (2006). Book Review: Goodman and Gilman’s The Pharmacological Basis of Therapeutics: Digital Edition, 11th Edition. Ann. Pharmacother..

[10] Fortuni B., Inose T., Ricci M., Fujita Y., Van Zundert I., Masuhara A., Fron E., Mizuno H., Latterini L., Rocha S. (2019). Polymeric Engineering of Nanoparticles for Highly Efficient Multifunctional Drug Delivery Systems. Sci. Rep..

[11] Zakeri A., Kouhbanani M.A.J., Beheshtkhoo N., Beigi V., Mousavi S.M., Hashemi S.A.R., Zade A.K., Amani A., Savardashtaki A., Mirzaei E. (2018). Polyethylenimine-based nanocarriers in co-delivery of drug and gene: A developing horizon. Nano Rev. Exp..

[12] Zhou B., Zhao L., Shen M., Zhao J., Shi X. (2017). A multifunctional polyethylenimine-based nanoplatform for targeted anticancer drug delivery to tumors in vivo. J. Mater. Chem. B.

[13] Ihm J.E., Krier I., Lim J.M., Shim S., Han D.K., Hubbell J.A. (2015). Improved biocompatibility of polyethylenimine (PEI) as a gene carrier by conjugating urocanic acid: In vitro and in vivo. Macromol. Res..

[14] Xia T., Kovochich M., Liong M., Meng H., Kabehie S., George S., Zink J.I., Nel A.E. (2009). Polyethyleneimine Coating Enhances the Cellular Uptake of Mesoporous Silica Nanoparticles and Allows Safe Delivery of siRNA and DNA Constructs. ACS Nano.

[15] Hilgenbrink A.R., Low P.S. (2005). Folate Receptor-Mediated Drug Targeting: From Therapeutics to Diagnostics. J. Pharm. Sci..

[16] Jones S.K., Sarkar A., Feldmann D.P., Hoffmann P., Merkel O. (2017). Revisiting the value of competition assays in folate receptor-mediated drug delivery. Biomaterials.

[17] Zwicke G.L., Mansoori G.A.H.A.G.A., Jeffery C.J. (2012). Utilizing the folate receptor for active targeting of cancer nanotherapeutics. Nano Rev..

[18] Lao J., Madani J., Puértolas T., Álvarez M., Hernández A., Pazo-Cid R., Ángel A., Torres A. (2013). A. Liposomal Doxorubicin in the Treatment of Breast Cancer Patients: A Review. J. Drug Deliv..

[19] Lovitt C.J., Shelper T.B., Avery V.M. (2018). Doxorubicin resistance in breast cancer cells is mediated by extracellular matrix proteins. BMC Cancer.

[20] Trebunova M., Laputková G., Slabá E., Lacjakova K., Verebova A. (2012). Effects of docetaxel, doxorubicin and cyclophosphamide on human breast cancer cell line MCF-7. Anticancer Res..

[21] Lawrie T.A., Rabbie R., Thoma C., Morrison J. (2013). Pegylated liposomal doxorubicin for first-line treatment of epithelial ovarian cancer. Cochrane Database Syst. Rev..

[22] Nguyen J., Solimando D.A., Waddell J.A. (2016). Carboplatin and Liposomal Doxorubicin for Ovarian Cancer. Hosp. Pharm..

[23] Pisano C., Cecere S.C., Di Napoli M., Cavaliere C., Tambaro R., Facchini G., Scaffa C., Losito S., Pizzolorusso A., Pignata S. (2013). Clinical Trials with Pegylated Liposomal Doxorubicin in the Treatment of Ovarian Cancer. J. Drug Deliv..

[24] Fukuokaya W., Kimura T., Miki J., Kimura S., Watanabe H., Bo F., Okada D., Aikawa K., Ochi A., Suzuki K. (2020). Effectiveness of Intravesical Doxorubicin Immediately Following Resection of Primary Non–muscle-invasive Bladder Cancer: A Propensity Score-matched Analysis. Clin. Genitourin. Cancer.

[25] Hong Y., Che S., Hui B., Yang Y., Wang X., Zhang X., Qiang Y., Ma H. (2019). Lung cancer therapy using doxorubicin and curcumin combination: Targeted prodrug based, pH sensitive nanomedicine. Biomed. Pharmacother..

[26] Prados J., Melguizo C., Cabeza L., Ortiz R., Caba O., Rama A.R., Delgado A.V., Arias J. (2015). Enhanced antitumoral activity of doxorubicin against lung cancer cells using biodegradable poly(butylcyanoacrylate) nanoparticles. Drug Des. Dev. Ther..

[27] Bey E.A., Bentle M.S., Reinicke K.E., Dong Y., Yang C.-R., Girard L., Minna J.D., Bornmann W.G., Gao J., Boothman D.A. (2007). An NQO1- and PARP-1-mediated cell death pathway induced in non-small-cell lung cancer cells by beta-lapachone. Proc. Natl. Acad. Sci. USA.

[28] Lee J.I., Choi D.Y., Chung H.S., Seo H.G., Woo H.J., Choi B.T., Choi Y.H. (2006). beta-lapachone induces growth inhibition and apoptosis in bladder cancer cells by modulation of Bcl-2 family and activation of caspases. Exp. Oncol..

[29] Lin M.T., Chang C.C., Chen S.-T., Chang H.-L., Su J.-L., Chau Y.-P., Kuo M.-L. (2004). Cyr61 expression confers resistance to apoptosis in breast cancer MCF-7 cells by a mechanism of NF-kappaB-dependent XIAP up-regulation. J. Biol. Chem..

[30] Chau Y.P., Shiah S.G., Don M.J., Kuo M.L. (1998). Involvement of hydrogen peroxide in topoisomerase inhibitor beta-lapachone-induced apoptosis and differentiation in human leukemia cells. Free Radic. Boil. Med..

[31] Shiah S.G., Chuang S.E., Chau Y.P., Shen S.C., Kuo M.L. (1999). Activation of c-Jun NH2-terminal kinase and subsequent CPP32/Yama during topoisomerase inhibitor beta-lapachone-induced apoptosis through an oxidation-dependent pathway. Cancer Res..

[32] Don M.J., Chang Y.H., Chen K.K., Ho L.K., Chau Y.P. (2001). Induction of CDK inhibitors (p21(WAF1) and p27(Kip1)) and Bak in the beta-lapachone-induced apoptosis of human prostate cancer cells. Mol. Pharm..

[33] Liu T.-J., Lin S.-Y., Chau Y.-P. (2002). Inhibition of poly(ADP-ribose) polymerase activation attenuates beta-lapachone-induced necrotic cell death in human osteosarcoma cells. Toxicol. Appl. Pharmacol..

[34] Lai C.C., Liu T.J., Ho L.K., Don M.J., Chau Y.P. (1998). beta-Lapachone induced cell death in human hepatoma (HepA2) cells. Histol. Histopathol..

[35] Li L.S., Bey E.A., Dong Y., Meng J., Patra B., Yan J., Xie X.-J., Brekken R.A., Barnett C.C., Bornmann W.G. (2011). Modulating endogenous NQO1 levels identifies key regulatory mechanisms of action of β-lapachone for pancreatic cancer therapy. Clin. Cancer Res..

[36] Middleton G.W., Ghaneh P., Costello E., Greenhalf W., Neoptolemos J.P. (2008). New treatment options for advanced pancreatic cancer. Expert Rev. Gastroenterol. Hepatol..

[37] Ough M., Lewis A., Bey E.A., Gao J., Ritchie J.M., Bornmann W., Boothman D.A., Oberley L.W., Cullen J.J. (2005). Efficacy of beta-lapachone in pancreatic cancer treatment: Exploiting the novel, therapeutic target NQO1. Cancer Boil. Ther..

[38] Thorn C.F., Oshiro C., Marsh S., Hernandez-Boussard T., McLeod H., Klein T.E., Altmana R.B. (2011). Doxorubicin pathways: Pharmacodynamics and adverse effects. Pharm. Genom..

[39] Silvers M.A., Deja S., Singh N., Egnatchik R.A., Sudderth J., Luo X., Beg M.S., Burgess S.C., DeBerardinis R.J., Boothman D.A. (2017). The NQO1 bioactivatable drug, β-Lapachone, alters the redox state ofNQO1+ pancreatic cancer cells, causing perturbation in central carbon metabolism. J. Biol. Chem..

[40] Giraudeau P., Baguet E. (2006). Improvement of the inverse-gated-decoupling sequence for a faster quantitative analysis of various samples by 13C NMR spectroscopy. J. Magn. Reson..

[41] Otte D.A.L., Borchmann R.E., Lin C., Weck M., Woerpel K.A. (2014). 13C NMR Spectroscopy for the Quantitative Determination of Compound Ratios and Polymer End Groups. Org. Lett..

[42] Cao X., Li Z., Song X., Cui X., Cao P., Liu H.-J., Cheng F., Chen Y. (2008). Core-shell type multiarm star poly(ε-caprolactone) with high molecular weight hyperbranched polyethylenimine as core: Synthesis, characterization and encapsulation properties. Eur. Polym. J..

[43] Li J., Xu S., Zheng J., Pan Y., Wang J., Zhang L., He X., Liu D. (2012). Polypeptide-based star-block quadripolymers as unimolecular nanocarriers for the simultaneous encapsulation of hydrophobic and hydrophilic guests. Eur. Polym. J..

[44] Liu H., Shen Z., Stiriba S.-E., Chen Y., Zhang W., Wei L. (2006). Core–shell-type multiarm star polyethylenimine-block-poly(ε-caprolactone): Synthesis and guest encapsulation potential. J. Polym. Sci. A Polym. Chem..

[45] Kasprzak A., Grudzinski I.P., Bamburowicz-Klimkowska M., Parzonko A., Gawlak M., Poplawska M. (2018). New insight into the synthesis and biological activity of the polymeric materials consisting of folic acid and *β*-cyclodextrin. Macromol. Biosci..

[46] Bekers O., Beijnen J., Otagiri M., Bult A., Underberg W. (1990). Inclusion complexation of doxorubicin and daunorubicin with cyclodextrins. J. Pharm. Biomed. Anal..

[47] Gidwani B., Vyas A. (2015). A Comprehensive Review on Cyclodextrin-Based Carriers for Delivery of Chemotherapeutic Cytotoxic Anticancer Drugs. BioMed Res. Int..

[48] Nasongkla N., Wiedmann A.F., Bruening A., Beman M., Ray D., Bornmann W.G., Boothman D.A., Gao J. (2003). Enhancement of solubility and bioavailability of β-lapachone using cyclodextrin inclusion complexes. Pharm. Res..

[49] Nakamura Y., Mochida A., Choyke P.L., Kobayashi H. (2016). Nanodrug delivery: Is the enhanced permeability and retention effect sufficient for curing cancer?. Bioconjugate Chem..

[50] Golombek S.K., May J.-N., Theek B., Appold L., Drude N., Kiessling F., Lammers T. (2018). Tumor targeting via EPR: Strategies to enhance patient responses. Adv. Drug Deliv. Rev..

[51] Hunter R.J. (1988). Zeta Potential in Colloid Science. Principles and Applications.

[52] Kratz F., Müller I.A., Ryppa C., Warnecke A. (2008). Prodrug Strategies in Anticancer Chemotherapy. ChemMedChem.

[53] Duncan R., Richardson S.C. (2012). Endocytosis and Intracellular Trafficking as Gateways for Nanomedicine Delivery: Opportunities and Challenges. Mol. Pharm..

[54] Li Z., Zhang Y., Zhu D., Li S., Yu X., Zhao Y., Ouyang X., Xie Z., Li L. (2017). Transporting carriers for intracellular targeting delivery via non-endocytic uptake pathways. Drug Deliv..

[55] Jain A.K., Panchagnula R. (2000). Review skeletal drug delivery systems. Int. J. Pharm..

[56] Kato Y., Ozawa S., Miyamoto C., Maehata Y., Suzuki A., Maeda T., Baba Y. (2013). Acidic extracellular microenvironment and cancer. Cancer Cell Int..

[57] Tannock I.F., Rotin D. (1989). Acid pH in tumors and its potential for therapeutic exploitation. Cancer Res..

[58] Ahmed S.A., Gogal R.M., Walsh J.E. (1994). A new rapid and simple non-radioactive assay to monitor and determine the proliferation of lymphocytes: An alternative to [3H] thymidine incorporation assay. J. Immunol. Methods.

[59] Costa P.C., Lobo J.M.S. (2001). Modeling and comparison of dissolution profiles. Eur. J. Pharm. Sci..

